# An Overview of Significant Achievements in Ruthenium-Based Molecular Water Oxidation Catalysis

**DOI:** 10.3390/molecules24030494

**Published:** 2019-01-30

**Authors:** Jayneil M. Kamdar, Douglas B. Grotjahn

**Affiliations:** Department of Chemistry and Biochemistry, San Diego State University; San Diego, CA 92182-1030, USA; jayneil.kamdar@gmail.com

**Keywords:** ruthenium, water oxidation, catalysis

## Abstract

Fossil fuels (coal, oil, natural gas) are becoming increasingly disfavored as long-term energy options due to concerns of scarcity and environmental consequences (e.g., release of anthropogenic CO_2_). Hydrogen gas, on the other hand, has gained popularity as a clean-burning fuel because the only byproduct from its reaction with O_2_ is H_2_O. In recent decades, hydrogen derived from water splitting has been a topic of extensive research. The bottleneck of the water splitting reaction is the difficult water oxidation step (2H_2_O → O_2_ + 4H^+^ + 4e^−^), which requires an effective and robust catalyst to overcome its high kinetic barrier. Research in water oxidation by molecular ruthenium catalysts enjoys a rich history spanning nearly 40 years. As the diversity of novel ligands continues to widen, the relationship between ligand geometry or electronics, and catalyst activity is undoubtedly becoming clearer. The present review highlights, in the authors’ opinion, some of the most impactful discoveries in the field and explores the evolution of ligand design that has led to the current state of the art.

## 1. Introduction

Technologies for harnessing wind, geothermal, hydropower, and solar energy are continually advancing and are beginning to play a formidable role in world energy production. Yet, despite the enormous amount of energy that can be generated, such renewable energy platforms suffer from the disadvantage of being greatly dependent on Mother Nature. For example, geothermal energy is limited to areas near tectonic plate boundaries. Wind energy is at the mercy of the whims of geographical weather patterns. Harnessing solar energy depends on hours of daylight in a geographical location or how sunny it is on any given day. Thus, it is important to find a way to efficiently store the energy harnessed by the new technologies so that it can be accessed anywhere and anytime. Historically our energy has been stored in chemical bonds. The C-H and C-C bonds of hydrocarbons in fossil fuels store energy derived from prehistoric photosynthetic mechanisms and this chemical energy is released as usable heat energy in a combustion reaction with oxygen. However, the sustainability of fossil fuel usage is becoming increasingly dubious with dwindling global supplies and atmospheric warming by carbon dioxide, a primary by-product of fossil fuel combustion.

From the 1950s to the 1970s, the discussion of distancing mankind from carbonaceous fuel inspired the idea of a “hydrogen economy”, which is based on the idea that the H-H bond of hydrogen gas can be used to store energy [1,2,3,4,5,6]. From an environmental standpoint, hydrogen is an attractive fuel because the only by-product from its reaction with O_2_ is pure H_2_O (Equation (1)):

2H_2_ (g) + O_2_ (g) → 2H_2_O (1)

Infrastructure for a hydrogen economy is expanding year by year with new hydrogen fueling stations emerging globally and major auto companies announcing the production of vehicles with fuel cell technologies (e.g., Toyota Mirai, Honda Clarity Fuel Cell, Toyota City and Tokyo, Japan, respectively). However, the critical problem with hydrogen remains that 95% of it is produced from fossil fuels by processes such as steam reforming, partial oxidation of methane, or coal gasification [7,8,9]. Thus, the hydrogen economy in its current state is not a carbon-neutral alternative:

2H_2_O (l) → 2H_2_ (g) + O_2_ (g) (2)

About 5% of global hydrogen is derived from water electrolysis (Equation (2)). Electrolysis is an attractive way to produce hydrogen because water is extremely abundant on the Earth’s surface and no greenhouse gases (e.g., CO_2_) are produced in the process. The challenge for widespread commercial adoption of this method lies in overcoming the inherent energetic costs associated with the reaction, which can be understood better by breaking down the reaction into its two half-reactions:

anode: →2H_2_O (l) → O_2_ (g) + 4H^+^ + 4e^−^ (3)

cathode: →4H^+^ + 4e^−^ → 2H_2_ (g) (4)

A 4 e^−^ oxidation of two water molecules generates O_2_ and protons at the anode (Equation (3)), while at the cathode, protons generated at the anode are reduced to produce H_2_ gas (Equation (4)). Considering only thermodynamics at first, under highly acidic conditions (pH ~ 0), the standard oxidation potential for Equation (3) is E° = 1.23 V and the standard reduction potential for Equation (4) is E° = 0.0 V. Raising the pH will vary the standard potentials according to the Nernst equation (ΔE = −59 mV/pH unit); however, the overall potential difference will remain ΔE = −1.23 V, which translates to an extremely endothermic reaction with a ΔG value of ~ + 475 kJ per mol of O_2_ formed. Thus, it is already apparent from a thermodynamic standpoint that splitting water is an uphill battle. In addition, one must overcome the kinetic barrier associated with each half reaction that manifests itself as “overpotential”. Appel and Helm defined “overpotential” as “the difference between the equilibrium potential for a given reaction (also called the thermodynamic potential) and the potential at which the catalyst operates at a specific current under specific conditions” [10]. From a molecular perspective, the anodic water oxidation half reaction is kinetically the more difficult step likely because not only does it require the efficient transfer of four electron and proton equivalents, but also the difficult formation of a weak O-O single bond must occur in the intermediate steps of the reaction. 

**Figure 1:**
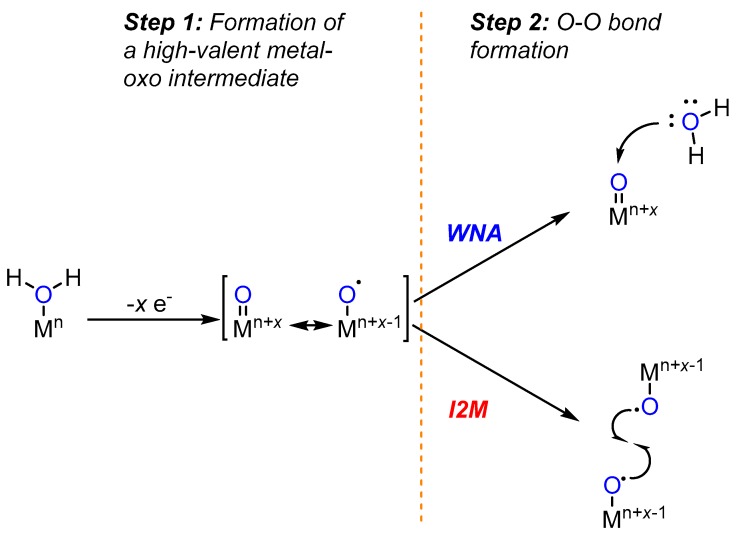
Prevalent mechanistic scenarios for metal catalysed oxidation of water.

Metals, particularly transition metals, can mediate electron movement during water oxidation and in turn mitigate the energetic cost associated with O-O bond formation. Two prevalent mechanistic scenarios discussed in the literature are: (1) water nucleophilic attack (WNA) and (2) bimolecular radical oxo-coupling (I2M) (Figure 1). Requisite to both scenarios is the generation of a high-valent metal oxo intermediate formed from an aquo ligand through successive proton-coupled electron transfers. The reactivity of the oxo group is then dictated by the d-electron count and the ligand field of the metal. In the WNA pathway, a second water molecule serves as a nucleophile to attack a highly electrophilic oxo ligand. The electrophilicity of the oxo is governed largely by the oxidation state of the metal and the molecular environment effected by the ligand geometry. From an orbital perspective, a σ bonding orbital of water overlaps with the anti-bonding π * orbital of the metal-oxo group leading to breakage of the double bond, formal reduction of the metal center by 2 e^−^, and O-O σ bond formation. In the I2M pathway, two metal-oxo fragments bearing radical character combine for a O-O bond formation. The combination of singly-occupied anti-bonding π * orbitals of the metal-oxo fragments leads to a O-O bonding interaction and reduction of bond order in the metal-oxo double bond [11].

## 2. A Brief History and Mini-review of Ru-Based Homogeneous Water Oxidation Catalysis

A growing number of transition metals, such as manganese [12], iridium [13], iron [14,15,16], copper [17], and cobalt [18,19,20,21,22] are known to make WOC but ruthenium, in particular, has received tremendous attention in the last 40 years. Interestingly, after a thorough literature search, it appears that the first account of oxidation of water by ruthenium ranges back as far as 1959 in a communication by Gortsema and Cobble [23]; remarkably, this paper has been cited only twice in the context of water oxidation. While details are sparse, it was reported that RuO_4_ was reduced to a Ru^IV^ species with either H_2_O_2_ or Fe^II^, and addition of an oxidizing agent (Ce^IV^ or Cr^VI^) in attempts to re-oxidize it back to RuO_4_ lead to some evolution of oxygen. Interest in ruthenium, however, really gained momentum after landmark studies by T. J. Meyer’s group between 1978 and 1981 showing that stable Ru-oxo complexes could be formed by proton-coupled electron transfer (PCET) [24,25,26]; these studies were followed by a seminal paper describing a dinuclear ruthenium complex, [(bpy)_2_(H_2_O)Ru(µ-O)Ru(H_2_O)(bpy)_2_]^4+^ (**1**) (bpy = 2,2′-bipyridine), also known as the “blue dimer”, that is considered to be the first characterized ruthenium-based homogeneous water oxidation catalyst [27]. The vast majority of Ru-based molecular catalysts are composed of some type of polypyridyl framework, hence a brief discussion on Ru-polypyridyl chemistry is an appropriate starting point. 

### 2.1. Ru-Polypyridyl Chemistry

Research on polypyridyl ruthenium complexes was spearheaded by Australian chemist Francis P. Dwyer along with several collaborators between the 1940s and 1960s. Dwyer and coworkers laid some of the important synthetic groundwork for making Ru and Os polypyridyl complexes. For example, motivated by a publication in 1955 by Brandt that identified mono- and bis-2,2′-bipyridine Ru^III^ complexes as intermediates in the formation for Ru^II^(bpy)_3_^2+^ [28], Dwyer reported procedures to intentionally prepare both mono- and bis-2,2′-bipyridine complexes [29]. Much of his early work also focused on exploring phenomena related to the optical activities of polypyridyl complexes. One important discovery was made in 1949: Dwyer and coworkers synthesized Ru^II^(phen)_3_^2+^ (phen = phenanthroline) and demonstrated that it maintained its optical activity upon oxidation to Ru^III^ and that a simple electrochemical change was achievable because of its substitutional “inertness” [30]. Indeed Ru-polypyridyl complexes tend to be more robust against substitution. From an electronic perspective, accessibility of the metal-ligand σ *(LUMO) orbitals is not as facile because they are destabilized by the relatively strong σ-donating ability of pyridyl ligands; metal-to-ligand back bonding into pyridyl π * orbitals drives ligand field splitting energies to increase even further. Moreover, polypyridyl ligands exhibiting a denticity of ≥ 2 are even more impervious to ligand substitution due to additional stability afforded by the chelate effect.

After Dwyer’s death in 1962, numerous collaborators continued work on the ligand substitution properties of polypyridyl Ru and Os complexes until the late 1960s. In the 1970s, Meyer picked up where Dwyer and his collaborators left off, investigating the novel synthetic chemistry of Ru/Os polypyridyl complexes, particularly their electron transfer properties and how redox reactions could potentially influence the chemical properties of coordinated ligands. For example, in one study, Meyer and coworkers found that primary amines coordinated to ruthenium as in [Ru^II^(bpy)_2_(NH_2_R)_2_]^2+^ were oxidized to nitriles upon oxidation of Ru^II^ to Ru^III^ [31]. Presumably based on analogous logic, Bruce Moyer, a graduate student of Meyer’s, prepared the Ru-aquo complex Ru^II^(bpy)_2_(py)(H_2_O)^2+^ with hopes that redox properties of a complex bearing a metal-coordinated water molecule could provide a pathway for the oxidation of water to O_2_ [24,32]. Results of the redox and chemical properties of Ru^II^(bpy)_2_(py)(H_2_O)^2+^ were described in a series of landmark studies from 1978 to 1981 (to be discussed in the following section), which have not only inspired modern Ru water oxidation chemistry but also became the basis of the theory of proton-coupled electron transfer (PCET). Polypyridyl ligands on Ru are attractive because they can withstand metal redox cycling (e.g., Ru^II^-Ru^VI^) and thus serve as rugged platforms for studying electrochemical/chemical phenomena on the metal center. In addition, the expansive available literature for pyridine functionalization is useful towards building a diverse library of strategically functionalized ligands to study the relationship between ligand electronics/geometry and catalytic activity.

### 2.2. A Stable Ru-Oxo Species Derived from a Ru-Aquo Complex: Ru^II^(bpy)_2_(py)(H_2_O)^2+^

**Figure 2:**
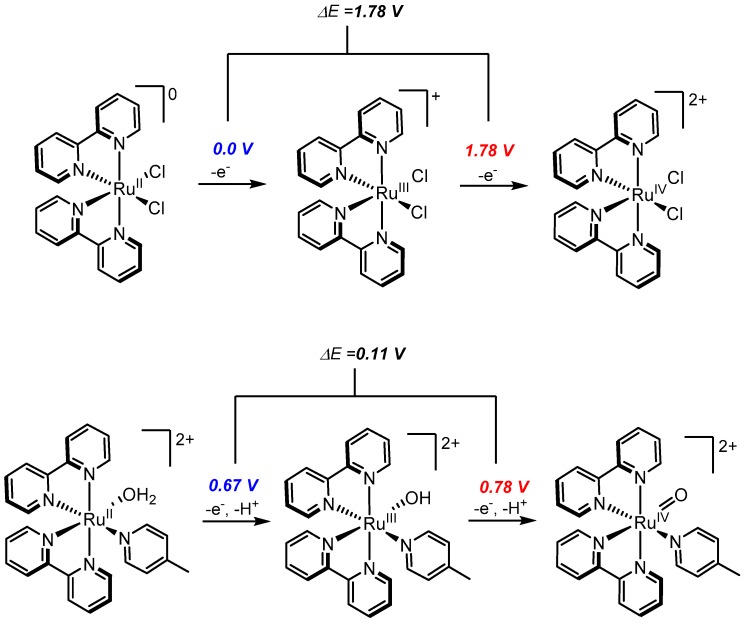
Work done by Meyer showing facile formation of a Ru-oxo intermediate through proton-coupled electron transfer (PCET).

A simple electrochemical comparison of the Ru^II/III^ and Ru^III/IV^ couples of Ru(bpy)_2_Cl_2_ and Ru(bpy)_2_(py)(H_2_O)^2+^ (py = pyridine) in pH 7 buffer reveals interesting differences (Figure 2) [25,33]. In the case of Ru(bpy)_2_Cl_2_, the Ru^II/III^ and Ru^III/IV^ couples are observed at 0.0 V and 1.7 V, respectively. In contrast, the Ru^II/III^ and Ru^III/IV^ couples for Ru(bpy)_2_(py)(H_2_O)^2+^ are observed at 0.67 V and 0.78 V, respectively. The dramatic difference between ΔE values (ΔE = 1.7 V for Ru(bpy)_2_Cl_2_ and ΔE = 0.11 V for Ru(bpy)_2_(py)(H_2_O)^2+^) indicates that the simple replacement of anionic Cl^−^ ligands with a neutral pyridine and an aquo ligand has a profound influence on redox activity. A Pourbaix diagram of Ru(bpy)_2_(py)(H_2_O)^2+^ shows that between pH ~0.85 to 10.25, the Ru^II/III^ couple is pH dependent (ΔE = 59 mV per pH unit) and therefore associated with concomitant transfer of a single proton. Similarly, between pH ~0.85 to 12, the Ru^III/IV^ couple is also associated with a single proton transfer [25]. Considering that the only reasonable choice of dissociable protons would be those of the aquo ligand, it can be deduced that a stepwise 2 e^−^/2 H^+^ transition occurs reversibly from [Ru^II^-OH_2_]^2+^ to [Ru^IV^=O]^2+^. The important implications of this discovery are the following: (1) PCET prevents excessive charge build-up that would normally disfavor the formation of Ru^IV^ species (as exemplified by the high Ru^III/IV^ oxidation potential of 1.78 V for Ru(bpy)_2_Cl_2_), and (2) PCET allows for the facile formation of stable Ru-oxo species (rather than through chemical reduction of RuO_4_) that can be exploited for further oxidative chemistry such as oxidation of water or organic substrates. 

### 2.3. The Blue Dimer–the First Homogeneous Ruthenium Water Oxidation Catalyst

**Figure 3:**
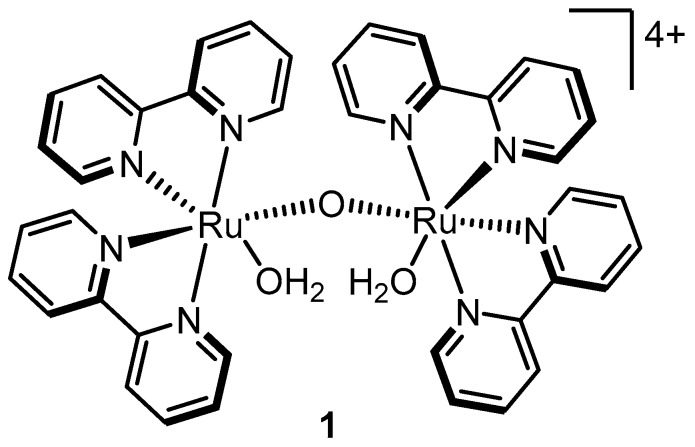
Molecular structure of the “blue dimer”.

Attempts by Meyer and coworkers to oxidize water with Ru(bpy)_2_(py)(H_2_O)^2+^ and other similar single-site Ru complexes lead to little or no success [25,33]. This observation lead to the presumption that more than one metal center was required to oxidize water, a logical conclusion at the time considering that the oxygen-evolution complex in the photosystem II was determined to contain several manganese atoms (although later both Thummel and Meyer showed that single-site water oxidation is indeed possible-see Section 2.6). The “blue dimer,” **1** (Figure 3), first synthesized in 1975, was a logical choice to test for catalytic activity because it contained two metal centers with aquo ligands in close proximity that could be transformed to oxo fragments by chemistry similar to that observed for Ru(bpy)_2_(py)(H_2_O)^2+^. Rapid O_2_ production was observed upon addition of 50–100 fold excesses of ceric ammonium nitrate (CAN) to a solution of **1** in 0.1 M HClO_4_. In addition, a large oxidative wave was observed at 1.20 V (vs. Ag/AgCl) by cyclic voltammetry of **1** in 0.1 M H_2_SO_4_, that was attributed to the catalytic oxidation of water [27]. 

### 2.4. An Anthracene-Bridged Dinuclear System—Electronically Isolated Metal Centers

**Figure 4:**
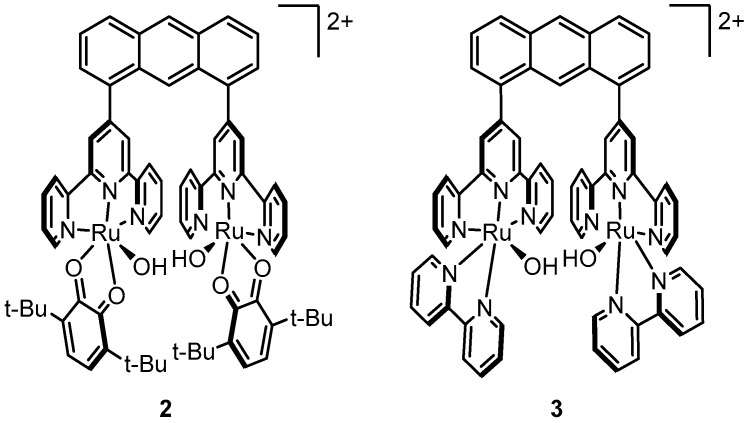
Anthracene-bridged dinuclear ruthenium complexes studied by Tanaka’s group.

Nearly 18 years after Meyer’s discovery of the blue dimer, Tanaka’s group reported **2**, a dinuclear complex shown in Figure 4 which consists of two (3,6-*t*Bu_2_qui)Ru^II^-OH (3,6-*t*Bu_2_qui = 3,6-di(*tert*-butyl)-1,2-benzoquinone) moieties connected by btpyan (1,8-bis{(2,2′:6′,2′′)-terpyridyl}anthracene), a bridging anthracene ligand [34]. Tanaka’s discovery was unique because it was a departure from the extensively explored µ-oxo motif following the report of the blue dimer [35,36,37,38,39,40,41,42,43,44,45,46,47,48,49,50,51,52,53,54,55]. In contrast to the blue dimer in which the Ru centers are electronically connected via the µ-oxo bridge, the two Ru centers in **2** are significantly more electronically isolated from each other due to the extended tpy-anthracene-tpy (tpy = 2,2′;6′,2′′-terpyridine) bridge of the ligand, yet the two metal centers are positioned closely enough to allow a Ru-O-O-Ru interaction to occur. The redox-active quinone ligands were shown to be reduced to semiquinones upon deprotonation of the Ru^II^-OH groups. Cyclic voltammetry of **2** deposited on an ITO electrode showed a ligand-centered redox wave at 0.40 V, an irreversible wave at 1.20 V corresponding to the Ru^II/III^ oxidation, and a significant catalytic wave with an onset potential of ~1.5 V. Controlled-potential electrolysis of **2** modified on ITO was conducted at +1.70 V in a pH 4 aqueous solution and oxygen evolution was observed for 40 h with a TON of 33,500 (quantified by GC). By contrast, under the same conditions, controlled-potential electrolysis of similar complex, [Ru(OH)(bpy)]_2_(btpyan)]^2+^ (**3**), lead to evolution of undetectable amounts of oxygen, suggesting that the redox-active quinone ligands of **2** are noninnocent in catalysis [56]. 

### 2.5. Dinuclear Catalysts with a more Rigid Backbone

**Figure 5:**
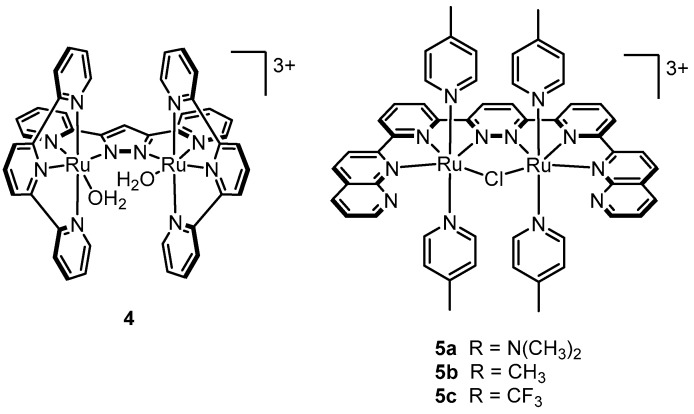
Dinuclear catalysts reported by Llobet’s (**4**) and Thummel’s groups (**5a**–**c**).

Llobet’s group discovered the dinuclear Ru-Hbpp catalyst (**4**, Figure 5) bearing a rigid 2,2′-(1*H*-pyrazole-3,5-diyl)dipyridine backbone [57]. Free rotation of the Ru-O moieties in **4** is restricted due to the rigidity of the Hbpp ligand and thus, unlike in complexes **1**–**3**, the Ru-O groups are intrinsically preorganized for intramolecular O-O bond formation via an I2M oxo-radical coupling pathway. An ^18^O-labelling study strongly indicated that O_2_ is indeed generated via an intramolecular radical coupling pathway [58]. Under acidic conditions (pH = 1) and excess Ce^IV^, turnover numbers close to 500 were obtained. Shortly after, Thummel and coworkers synthesized related dinuclear µ-Cl complexes (**5a**–**c**) with a rigid polypyridyl backbone positioning two Ru centers in close proximity [59]. Turnovers achieved by complexes **5a**–**c** were nearly two times that using complex **4**. Interestingly, the µ-Cl bridge appears to be very stable, to the extent that no ionization was observed even in a refluxing solution of Ag^+^ in acetone, although H_2_O/Cl^−^ substitution may be more likely upon oxidation in acidic aqueous conditions. The possibility of the Cl^−^ bridge remaining intact during the catalytic cycle cannot be ruled out until more thorough mechanistic studies are done.

### 2.6. One Metal Site Is Enough

As described above, the early years of the field of transition-metal WOC were dominated by the paradigm that a single metal site was insufficient to catalyze the oxidation of H_2_O to O_2_ because the multi-electron nature of the reaction could not be efficiently mediated by a single metal atom. 

**Figure 6:**
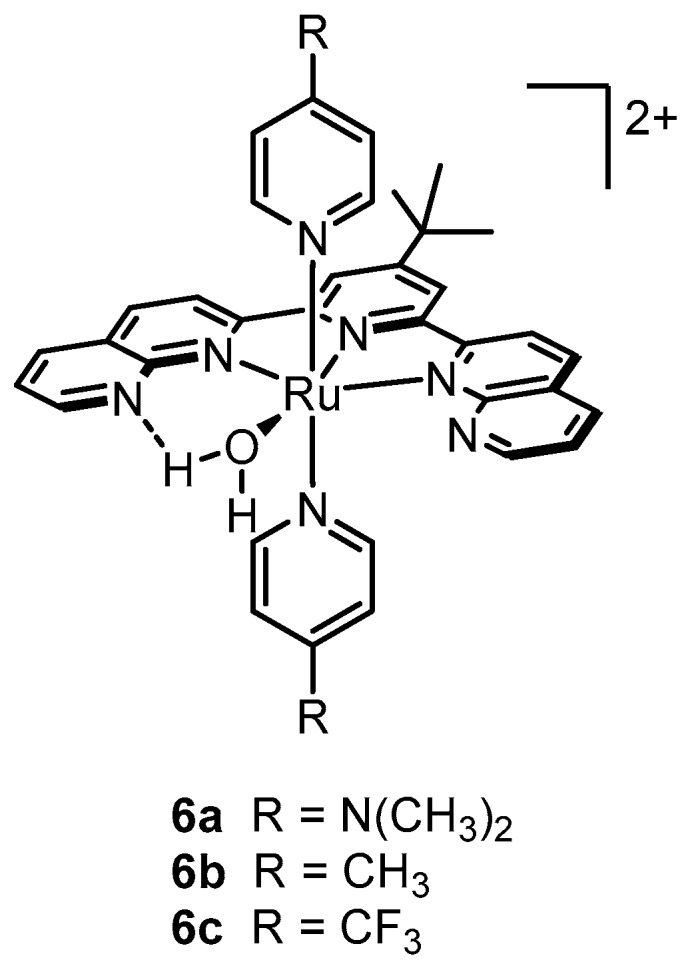
First mononuclear catalysts reported to oxidize water at rates comparable to previously reported dinuclear systems.

This belief was largely motivated by structural elucidation of the oxygen-evolving complex in photosystem II that revealed a multi-metallic Mn cluster as the active catalyst. The success achieved by the blue dimer contrasted with the poor or non-existent catalytic activity of mono-nuclear Ru complexes [25,60,61,62,63,64,65,66,67] further propagated the perception that at least two metal sites were necessary to support the multiple oxidizing equivalents required for water oxidation. However, in 2005, Zong and Thummel reported competent water oxidation by a series of mono-nuclear Ru complexes bearing a 2,6-di(1,8-naphthyridin-2-yl)pyridine backbone, a bound water molecule, and varying pyridyl axial groups (**6a**–**c**, Figure 6) [59]. 

The catalytic activities of **6a**–**c** were evaluated by measuring O_2_ evolution after injecting a solution of each catalyst dissolved in acetonitrile into an aqueous solution containing excess CAN (pH 1). Turnover numbers, particularly for **6b**, were comparable to previously reported di-nuclear Ru complexes, thus giving credibility for the first time to the idea that mono-nuclear Ru complexes are indeed capable of providing the oxidizing equivalents necessary for water oxidation. 

**Figure 7:**
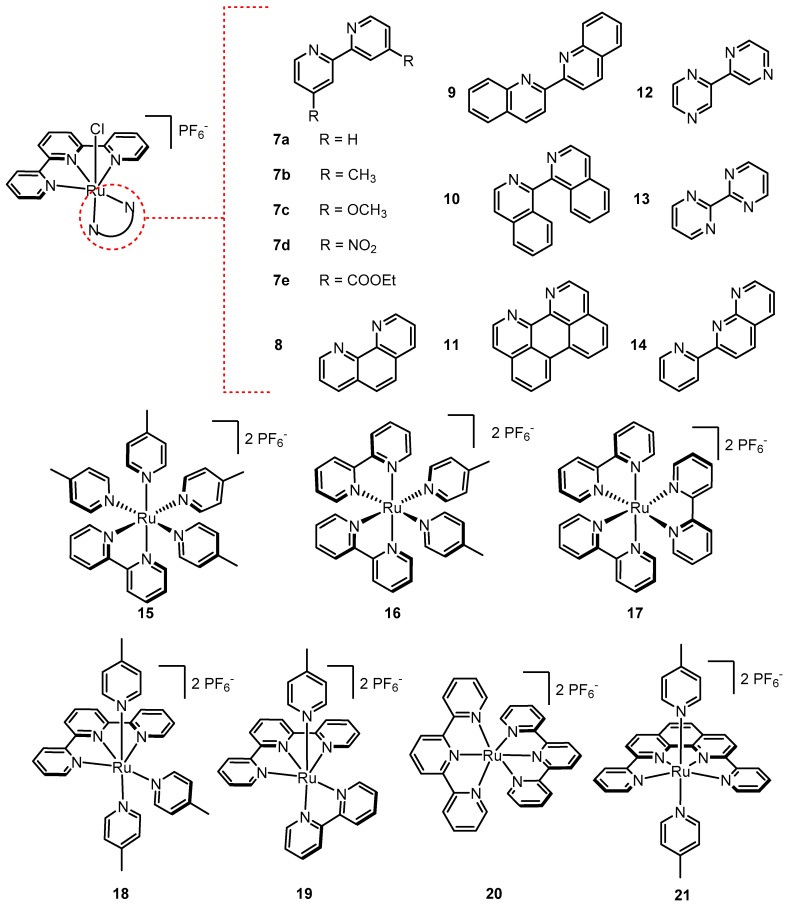
The series of mononuclear Ru-polypyridyl complexes tested by Thummel’s group for catalytic activity in the presence of Ce^IV^.

In 2008, Thummel’s group followed up with a systematic study of the catalytic activities of numerous mononuclear Ru complexes with various configurations of polypyridyl ligands (Figure 7) [68]. The first group of mononuclear complexes were based on [Ru(tpy)(bpy)Cl]^+^ using bpy derivatives (**7a**–**e**) and other related polypyridyl bidentate ligands (**8**–**14**). Complexes 7a–e and **8** were modestly active showing turnover numbers ranging from 110–570 under the authors’ conditions. Interestingly, with the exception of **14**, no oxygen evolution was detected (within the sensitivity of the authors’ method) from the remaining complexes (**9**–**13**). The second group of complexes (**15**–**21**) has pyridyl ligands in all six coordination sites; however the denticities of the ligands were systematically varied (pyridine, bipyridine, terpyridine, quaterpyridine). Complexes **15**–**17** and **20** showed no water oxidation activity while moderate turnover numbers were achieved by **18** and **19** (95 and 135 TON respectively). One complex clearly stands out: **21** contains 2,9-di(pyrid-2′-yl)-1,10-phenanthroline as a tetradentate ligand with 4-picoline ligands in the axial positions; 416 turnovers were achieved with a rate of 0.330 µmol of O_2_ min^−1^ (TOF = 27.5 × 10^−6^ s^−1^). Details on **21** and related derivatives will be discussed in Section 2.7. 

**Figure 8:**
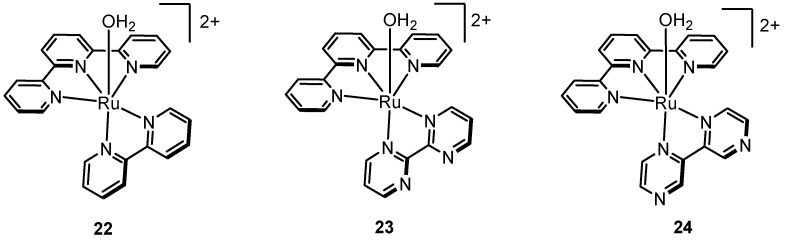
Aquated Ru(tpy)(NN) complexes that have played an important role in mechanistic elucidation of water oxidation at Ru.

Meanwhile, complementary work on complex **7a** by Sakai’s group revealed a significant induction period prior to the onset of water oxidation in the presence of excess Ce^IV^ [69]. In contrast, a maximum initial rate of O_2_ evolution and no induction period was observed from complex **22**, the aquated analog of **7a**, suggesting that the Ru-Cl complexes are not active water oxidation catalysts, but rather they serve as pre-catalysts that undergo Cl^−^/H_2_O substitution to generate the catalytically active Ru-OH_2_ analogs [69]. Wasylenko et al. monitored changes in the ^1^H NMR spectrum of **7a** in D_2_O and found that nearly 55% of **7a** was converted to the Ru-OD_2_ analog in 3 h [70]. Later in 2012, Thummel conducted a comparative study between **7a** and its halide counterparts, [Ru^II^(tpy)(bpy)Br]^+^ and [Ru^II^(tpy)(bpy)I]^+^ and found that the initial rate of [Ru^II^(tpy)(bpy)I]^+^ was similar to that of [Ru^II^(bpy)(tpy)(OH_2_)]^+^ (**22**, Figure 8) with no induction period [71]. The lack of an induction period led to speculation that the active species could be a seven-coordinate [Ru(I)(OH_2_) intermediate in which the Ru-I bond is retained during catalysis; however, Yan et al. elucidated this peculiarity by EPR and X-ray absorption spectroscopy showing that the Ru-I bond in fact undergoes facile dissociation upon oxidation to Ru^III^ leading to formation of the active aquo-complex [72]. 

**Figure 9:**
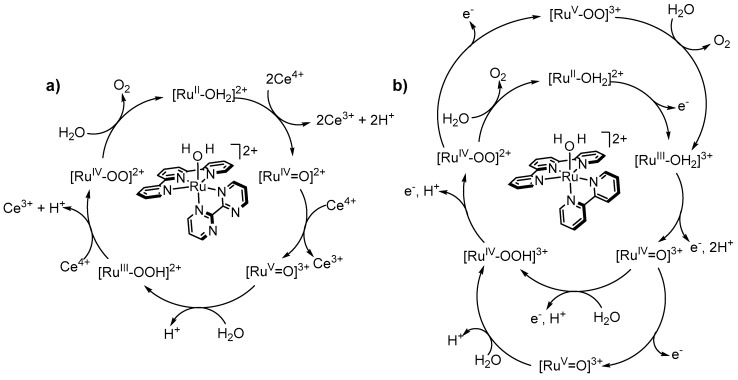
(**a**) Ce^IV^-mediated mechanism proposed by Meyer’s group of water oxidation by Ru(tpy)(bpy) complexes, (**b**) mechanism proposed by Pushkar.

Nearly coincident to Thummel’s and Sakai’s work on single site Ru-polypyridyl catalysts in 2008, Meyer’s group reported the first mechanistic study of two single-site Ru catalysts, [Ru(tpy)(bpm)(OH_2_)]^2+^ (**23**) and [Ru(tpy)(bpz)(OH_2_)]^2+^ (**24**) (Figure 8), that are aquated versions of **12** and **13**, respectively [73]. A general mechanism for these complexes was proposed based on electrochemical data and UV-visible spectroscopy (Figure 9a). Potential vs. pH dependence studies of **23** show a 2 e^−^/2 H^+^ change from pH 0 to pH 9.7 corresponding to the transition from [Ru^II^-OH_2_]^2+^ to [Ru^IV^=O]^2+^, and a 2 e^−^/1 H^+^ change from pH 9.7 to pH 14 corresponding to the transition from [Ru^II^-OH]^2+^ to [Ru^IV^=O]^2+^. The [Ru^IV^=O]^2+^ to [Ru^V^=O]^2+^ transition is pH independent at least from pH 0 to pH 3 (no data were shown past pH 3) and the ensuing large increase in current suggested that water oxidation is triggered upon reaching the Ru^V^ state. Reinforcing the notion that Ru^V^ plays a role, changes in UV-visible spectral patterns monitored upon addition of three equivalents of Ce^IV^ to a solution of [Ru^II^(tpy)(bpm)(OH_2_)]^2+^ suggested formation of a transient [Ru^V^=O]^3+^ species followed by a peroxido [Ru^III^-OOH]^2+^ species, which decomposed in a matter of minutes back to [Ru^II^-OH]^2+^. Under catalytic conditions (an excess of 30 equiv of Ce^IV^), the resting state appeared to be a [Ru^IV^-OO]^2+^ species where peroxido coordination may be bidentate. 

In 2014, Pushkar et al. asserted that the available evidence (from electrochemistry and UV-visible spectroscopy) for formation of a [Ru^V^=O]^3+^ species in Ru(tpy)(bpy)-type complexes was insufficient [74]. The authors presented spectroscopic data (EPR, X-ray absorption) supported by computations to suggest that [Ru^V^=O]^3+^ may in fact be absent in the mechanistic cycle of **22**. Addition of 20 equiv of Ce^IV^ to **22** and freezing within 30 sec after mixing resulted in a largely EPR silent species (~95%) ruling out paramagnetic Ru^III^ or Ru^V^ as the major species. In addition, XANES spectra showed a significant shift to higher energy consistent with Ru^IV^, and the Ru-O distance calculated from EXAFS measurements (1.82 Å) is consistent with a Ru^IV^=O species. The residual EPR signal (~5%) did not have the characteristic *g*-tensor of a Ru^V^=O species; based on DFT predictions, it was assigned as a Ru^III^-OOH peroxide alternative. The results of Pushkar et al. complement electrospray ionization mass spectrometric (ESI-MS) analyses by the Berlinguette group in which no signals for a [Ru^V^=O]^3+^ species were observed after addition of 3–4 equiv of Ce^IV^ or even under catalytic conditions (16 equiv of Ce^IV^) [75]. Furthermore, only minor spectroscopic changes were observed upon addition of 1 equiv of Ce^IV^ to the [Ru^IV^=O]^2+^ species, implying that the dominant species is likely [Ru^IV^=O]^2+^. 

The kinetics of O_2_ evolution by **22** have been studied in the presence of Ce^IV^. Meyer’s and Berlinguette’s groups separately reported O_2_ evolution to be first order with respect to [Ru] and zeroth order with respect to [Ce^IV^] when 30–200 equiv of Ce^IV^ were used, suggesting that electron transfer is not rate-limiting while chemical steps such as O-O bond formation or O_2_ release may be rate-limiting [73,75]. However, Masaoka and Sakai reported O_2_ evolution to be first order with respect to both [Ru] and [Ce^IV^] when 10 equiv of Ce^IV^ were used [69]. As Yagi et al. pointed out, O_2_ evolution with respect to Ce^IV^ likely operates under Michaelis-Menten-like kinetics where the rate law is dictated by the concentration of oxidant [76]. It has yet to be conclusively determined whether the rate-limiting step is O-O bond formation or O_2_ release in the presence of excess Ce^IV^. 

### 2.7. Emergence of Tetradentate Motif

**Figure 10:**
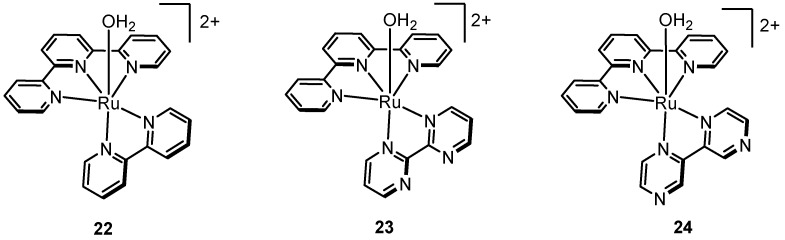
(**a**) Depiction of a common scenario of *cis*-*trans* isomerism in Ru(bpy)_2_(L)_2_ complexes where the *cis* configuration is favored, (**b**) utilization of a tetradentate ligand as a strategy to prevent isomerization.

**Figure 11:**
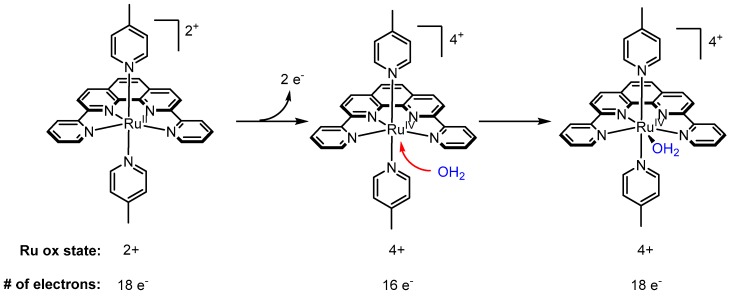
Thummel’s hypothesis on the mechanism of water oxidation by **21**.

Work done in the 1980s and 1990s demonstrated that *trans*-[Ru(bpy)_2_L_2_]^2+^ complexes are often prone to isomerization to their *cis* counterparts, thus making it difficult to study or exploit the chemical/photochemical properties of *trans* complexes (Figure 10a) [77,78,79,80]. One strategy to minimize the likelihood of a *trans*-to-*cis* conversion is to covalently link the two bipyridine ligands using a linker short enough to highly disfavor the *cis* configuration. The simplest, promising analog for such a strategy is the ligand 2,2′:6′,2′′:6′′,2′′′′-quaterpyridine (qtpy), which links two bipyridine units through a single C(sp^2^)-C(sp^2^) covalent bond (Figure 10b). Ruthenium quaterpyridine complexes were first reported by Che and coworkers in 1994, including *trans*-[Ru^II^(qtpy)(OH_2_)_2_]^2+^ and a crystal structure for *trans*-[Ru^II^(L)(PPh_3_)_2_[ClO_4_]_2_ (L = 3′′,5,5′,5′′′′-tetramethyl-2,2′:6′,2′′:6′′,2′′′′-quaterpyridine) [81]. The crystal structure shows that despite significant angle strain, the quaterpyridine ligand coordinates through all four binding sites, adopting a highly distorted square planar configuration where the N_1_-Ru-N_2_ angle involving the terminal pyridine groups is as wide as ~123°. One problem that is often encountered with quaterpyridine is rotation around the central C(sp^2^)-C(sp^2^) bond, which leads to the formation of a dinuclear species (not shown), where each half of the ligand separately participates in bidentate coordination to a single ruthenium atom [82]. To overcome this problem, Thummel’s group synthesized 2,9-di-(2′-pyridyl)-1,10-phenanthroline (dpp), in which the rigid phenanthroline moiety precludes any rotation around the central C(sp^2^)-C(sp^2^), and tetradentate coordination to ruthenium occurs readily [82]. As mentioned in Section 2.6., complex **21** performed better than most of the other polypyridyl Ru complexes tested with a TON of 416 and at the rate of 0.330 µmol of O_2_ min^−1^ (TOF = 27.5 × 10^−6^ s^−1^) in the presence of excess Ce^IV^ [68]. Unlike [(tpy)(bpy)Ru^II^-OH_2_]^2+^-type complexes where an aquo ligand is pre-coordinated in the first coordination sphere and available to participate in the ensuing catalytic cycle, complex **21** is coordinatively saturated with polypyridyl ligands. Thummel hypothesized that a Ru^IV^ 16 e^−^ complex, after loss of 2 e^−^, would be sufficiently electrophilic to accommodate metal-coordination of a water molecule as a seventh ligand to form an 18 e^−^ Ru^IV^(OH_2_) pentagonal bipyramidal intermediate and the wide N-Ru-N angle of **21** may be conducive to such a pathway (Figure 11). This hypothesis was supported by geometry optimization calculations where dissociation of water was observed in Ru^II^-OH_2_ and Ru^III^-OH_2_ complexes but not Ru^IV^-OH_2_ [68].

### 2.8. Anionic Ligands Stabilize Higher Oxidation States

Thummel previously demonstrated that electron-donating groups on pyridyl ligands could lower oxidation potentials [59,68,82,83,84]. Anionic ligands, in contrast to neutral ligands, can stabilize higher metal oxidation states even more effectively and dramatically lower redox potentials. 

**Figure 12:**
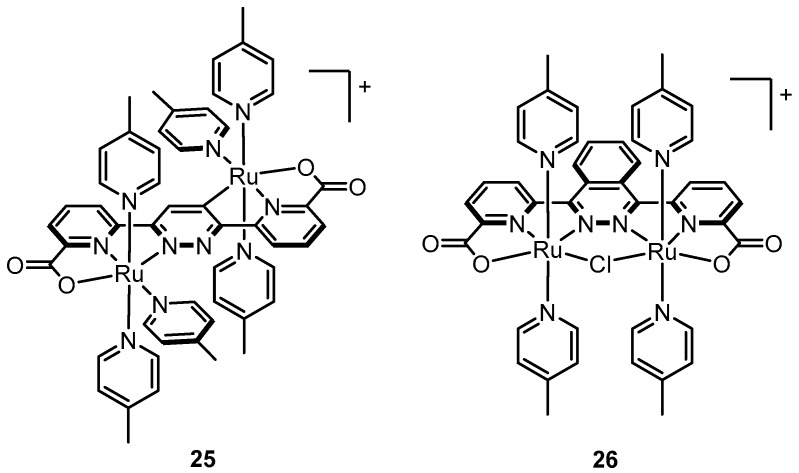
Dinuclear Ru catalysts with anionic groups.

In the context of water oxidation, Akermark and Sun first demonstrated this phenomenon in 2002 by comparison of two similar dinuclear Mn complexes—one with neutral pyridyl ligands and the other with anionic phenolates [85]. A combination of electrochemistry and EPR revealed significantly negative shifts in redox potentials for the complex bearing anionic ligands (e.g., ∆E = ~ −0.820 V for Mn^II^/Mn^II^ to Mn^II^/Mn^III^ and ∆E = ~ −1.02 V for Mn^II^/Mn^III^ to Mn^III^/Mn^III^). Akermark and Sun further applied this strategy to Thummel’s dinuclear Ru complex **5** by designing a ligand with terminal carboxylates rather than pyridyl functionalities [86]. Attempts to metalate the ligand resulted in complex **25** (Figure 12) that has an anti-configuration where the Ru centers are connected at opposing sides of the pyridazine ring, one with a C-Ru bond, the other with a N-Ru bond. Significantly, the oxidation potentials for **25** were dramatically lowered compared to **5** presumably due to the presence of anionic ligands, the two carboxylates and the unintended carbon ligand. In a subsequent report in 2010, Akermark and Sun reported Ru complex **26** with a similar ligand where the pyridazine ring was replaced by a phthalazine moiety to promote syn coordination, where the two ruthenium centers are adjacent to each other, and thus allow for a more appropriate comparison with **5** [87]. Interestingly, under the same conditions ([Ce^IV^] = 20 mM, [catalyst] = 0.1–0.5 µM), the rate for **26** was nearly four times greater than that observed by **25**.

### 2.9. Catalysts Bearing 2,2’-Bipyridine-6,6’-Dicarboxylate as Tetradentate Ligands

**Figure 13:**
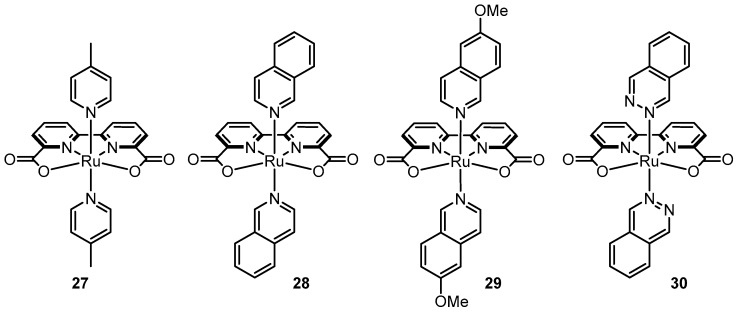
Prominent examples of Ru(bda) complexes with varying heterocycles in the axial positions.

**Figure 14:**
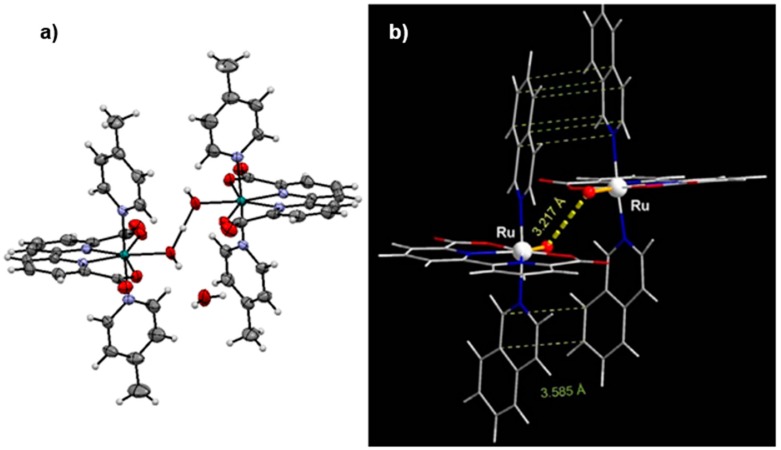
(**a**) X-ray crystal structure derived from crystals grown after addition of 2 equiv of CAN to **29** in the presence of NH_4_PF_6_. Adapted with permission from [88]. Copyright (2009) American Chemical Society. (**b**) Calculated “encounter complex” of two Ru^V^ = O molecules with π-stacking interactions between the four isoquinoline units. Reprinted by permission from Springer Nature: Nature Publishing Group, Nature Chemistry, A molecular ruthenium catalyst with water-oxidation activity comparable to that of photosystem II, L. Duan, F. Bozoglian, S. Mandal, B. Stewart, T. Privalov, A. Llobet, L. Sun, 2012 [90].

With the success achieved from incorporating anionic ligands into dinuclear Ru systems, Sun’s group applied the same tactic to complexes with tetradentate ligands such as **21**. A pivotal moment in the field was the discovery of complex (**27**, Figure 13) with 2,2’-bipyridine-6,6′-dicarboxylate (bda) as a tetradentate ligand, which bears similarity to **21**; however, the phenanthroline subunit is substituted with bipyridine and anionic carboxylates are incorporated in place of the terminal pyridyl groups [88]. X-ray crystallography of **27** confirmed tetradentate chelation of bda, and a wide O-Ru-O angle of 122.9° similar to the N-Ru-N angle of **21**. A side-by-side comparison of electrochemical data of **21** and **27**, shows that redox potentials of **27** are considerably more negative over a wide pH range [89]. For example, at pH 1, the Ru^II/III^ transition is nearly ~0.5 V more negative for **27** (E_1/2_ = ~0.6 V) compared to **21** (E_1/2_ = ~1.1 V). Similarly at pH 7, the Ru^II/III^ and Ru^III/IV^ transitions occur at 0.55 V and 0.80 V, respectively, for **27** and the 2 e^−^ Ru^II/IV^ transition for **21** occurs at 0.85 V. Furthermore, the onset potential for the catalytic oxidation wave occurs at ~1.55 V for **21** and at ~1.05 V for **27**, nearly ~0.5 V more negative and representing the lowest observed overpotential in the field thus far. Sun and coworkers also acquired a fascinating crystal structure of a seven-coordinate Ru^IV^-hydroxo intermediate from crystals grown after addition of 2 equiv of CAN to **27** and precipitation in the presence of excess NH_4_PF_6_ (Figure 14a) [88]. The crystal structure gives credence to the theory of a seven-coordinate Ru^IV^ intermediate initially proposed by Thummel with respect to complex **21**. The wide O-Ru-O angle may encourage coordination of a water molecule as a seventh ligand (discussed further below).

In another astounding discovery, Sun and Llobet showed that replacement of the axial 4-picoline ligands with isoquinoline ligands (**28**) lead to an improvement in catalytic rate by nearly an order of magnitude [90]. The second order kinetics of O_2_ evolution with respect to catalyst concentration point to an I2M pathway and DFT calculations suggest that greater π-stacking between isoquinoline units of two approaching molecules facilitates O-O bond formation (Figure 14b). Richmond et al. showed that implementation of isoquinoline units with a methoxy group (MeO-isoq) on the C-6 carbon (**29**) led to even greater turnover frequencies (about 1.5 to 3 times greater than for **28** under identical Ce^IV^ conditions) [91]. Structural analysis of calculated dimeric [Ru-O˖˖˖O-Ru]^2+^ transition states exhibited shorter distances for **29** compared to **28** due to increased π –stacking from a stacking geometry that favors an electrostatic interaction between a positively charged C-6 atom on one MeO-isoquinoline half and a negatively charged C atom on the other half. Furthermore, catalyst **30** with two axial phthalazine ligands was found to have very high turnover number (>50,000) consistent with calculations that show a directly proportional correlation between the longevity of catalysis and the HOMO energy of the axial ligand [92].

**Figure 15:**
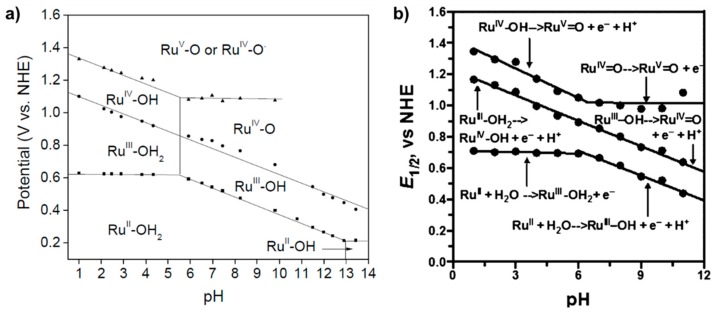
(**a**) Pourbaix diagram of **27** constructed by Sun’s group. Reprinted by permission from Springer Nature: Nature Publishing Group, Nature Chemistry, A molecular ruthenium catalyst with water-oxidation activity comparable to that of photosystem II, L. Duan, F. Bozoglian, S. Mandal, B. Stewart, T. Privalov, A. Llobet, L. Sun, 2012 [90]. (**b**) Pourbaix diagram of **28** constructed by Meyer’s group [93].

Sun’s group performed E vs. pH studies and constructed a Pourbaix diagram of **27** (Figure 15a). From pH 1 to ~5.5, E_1/2_ of Ru^II/III^ remains unchanged signifying a simple 1 e^−^ transfer; however PCET is observed for Ru^III/IV^ and Ru^IV/V^. At pH > ~5.5, PCET is observed for Ru^II/III^ and Ru^III/IV^ however the potential remains unchanged for Ru^IV/V^. They proposed that between pH 1 to ~5.5, formation of the Ru-OH_2_ complex occurs as early as the Ru^II^ oxidation state; however, the two aquo protons are lost with the Ru^III/IV^ and Ru^IV/V^ transitions and the Ru^II/III^ transition only involves electron transfer. At pH > ~5.5, they proposed two PCET steps from Ru^II^-OH_2_ to Ru^IV^=O followed by a simple electron transfer to Ru^V^=O. The E vs. pH dependence of **28**, independently investigated by Meyer’s group, closely resembles that of **27** by Sun’s group, suggesting that both catalysts operate fundamentally by a similar mechanism (Figure 15b) [93]. In contrast to Sun’s interpretation however, Meyer proposed that between pH 1 to ~5.5, water coordination does not occur until oxidation to Ru^III^ allowing two sequential PCETs to occur from Ru^III^-OH_2_ to Ru^V^ = O. At pH > ~5.5, the basicity of the cell solution is great enough to promote immediate proton transfer upon formation of Ru^III^-OH_2_. 

In addition to electrochemistry, EPR spectroscopy and X-ray absorption spectroscopy have played a complementary role in deciphering mechanistic details of the Ru(bda)-type catalysts. A recent study provided EPR evidence of a Ru^III^ seven-coordinate species [94]. Empirical evidence of the (bda)Ru^V^ = O intermediate is difficult to attain because the rate of subsequent O-O bond formation by the I2M mechanism is highly rapid. Studies have shown that the immobility of surface-bound Ru(bda) complexes relegates O-O bond formation to the WNA pathway, which is less efficient particularly under acidic conditions. Lebedev et al [95] exploited the catalytic inefficiency of a Ru(bda) complex bound to an ITO surface through phosphonate anchoring groups to trap and characterize a seven-coordinate Ru^V^=O intermediate [96]. Under acidic conditions, EPR spectra after electrolysis at 0.9 V and 1.7 V show distinct signals for Ru^III^ and Ru^V^, respectively. Holding the electrode potential at 1.5 V or 1.7 V, the resulting Ru^V^=O species was characterized by extended X-ray absorption fine structure (EXAFS) analysis and determined to have a Ru=O bond distance of 1.75 ± 0.02 Å consistent with DFT calculations for a seven-coordinate (bda)Ru^V^=O intermediate. 

### 2.10. External Bases Accelerate Catalysis

**Figure 16:**
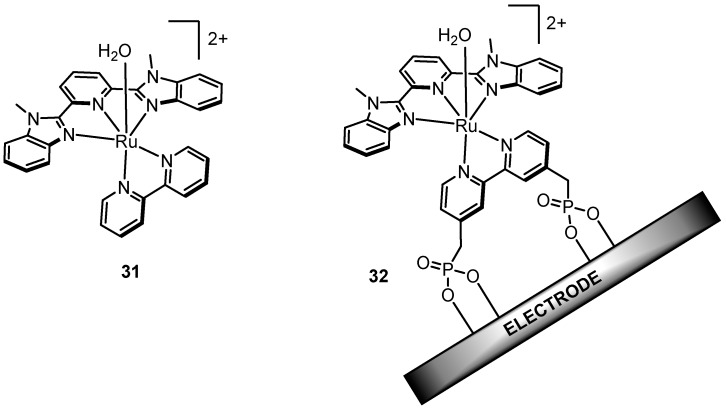
Complexes used by Meyer to show enhanced catalytic activity upon adding external bases.

**Figure 17:**
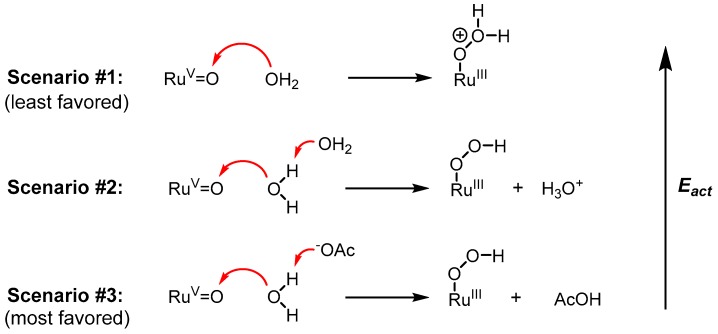
Relative energetics of an O-O bond formation by WNA.

In 2010, Meyer’s group made an important discovery in the field showing that electrocatalytic water oxidation, specifically the O-O bond formation step in WNA, is significantly enhanced by the addition of external bases [97]. Studies to explore this phenomenon were performed on [Ru(Mebimpy)(bpy)(OH_2_)]^2+^ (**31**, Figure 16**)** in solution and its related phosphonate derivative {Ru(Mebimpy)[4,4’-((OH)_2_OPCH_2_)_2_bpy]OH_2_)}^2+^ (**32**) covalently functionalized on an indium-tin oxide (ITO) electrode. Cyclic voltammetry was conducted on **31** and **32** in the presence of various external bases such as H_2_PO_4_^−^, OAc^−^, and HPO_4_^2−^_,_ where the ionic strength was kept constant at 0.1 M with appropriate addition of KNO_3_, and in all cases the data show an increase in catalytic current density (*i_cat_*) when compared to cyclic voltammetry performed with no added bases. Moreover, *i_cat_* appeared to increase as a function of base concentration and base strength, consistent with a base-associated rate dependence. The experimental observations were corroborated with quantum mechanical calculations on **31**. The lowest energy pathway calculated for O-O bond formation involves another water molecule in the secondary coordination sphere (Figure 17, Scenario #2) that works to solvate the proton released upon water-nucleophilic attack. Computations done without the proton-solvating water molecule lead to a highly charged and energetically unfavorable hydrogen peroxide intermediate [Ru^III^(Mebimpy)(bpy)(OOH_2_)]^3+^ (Figure 17, Scenario #1), demonstrating the necessity of the secondary water molecule. Analogous calculations with an acetate anion instead of a water molecule as the external base (Figure 17, Scenario #3) resulted in an even lower activation barrier (7.6 kcal/mol vs. 10.4 kcal/mol), consistent with experimental results. 

Meyer’s group published another study in 2015 exploring the effect of external bases on water oxidation by complex **28** [93]. The observed trend was similar to the 2010 study, where the rate of water oxidation increased as a function of base strength and base concentration. Two separate mechanistic scenarios were presented: (1) a concerted atom-proton transfer mechanism similar to the one described in Figure 17 (Scenario #3) where base-assisted water nucleophilic attack on a [Ru^V^=O]^+^ is the rate-limiting step, or (2) a mechanism where PCET from [Ru^IV^-OH]^+^ to [Ru^V^=O]^+^ is rate-limiting while the O-O bond formation step via bimolecular radical coupling is rapid. In each scenario, the proton transfer plays a critical role.

### 2.11. Internal Bases Accelerate Catalysis

Proton transfer is critical to accelerating the rate of water oxidation. One strategy to optimize proton transfer in a catalytic cycle is to incorporate basic sites internally within the ligand architecture in close proximity to the active site. Such a strategy has been implemented in the context of organic transformations and yielded impressive results [98,99,100,101]. 

**Figure 18:**
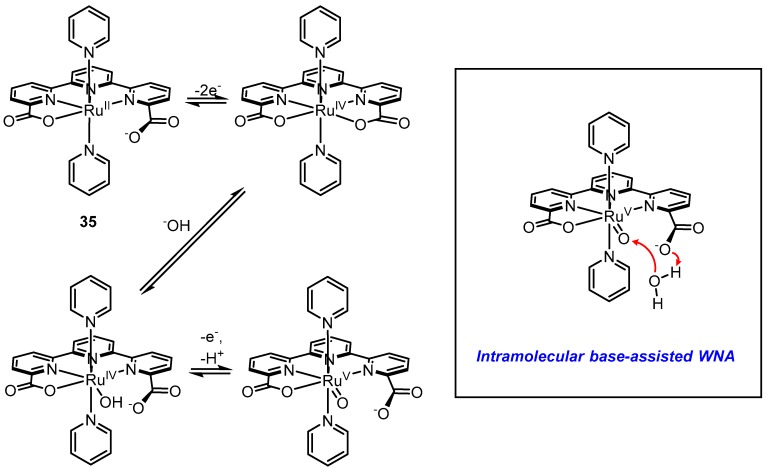
Proposed mechanism of water oxidation by [Ru^II^(tda-κ^4^-N_3_O)(py)] (**35**).

**Figure 19:**
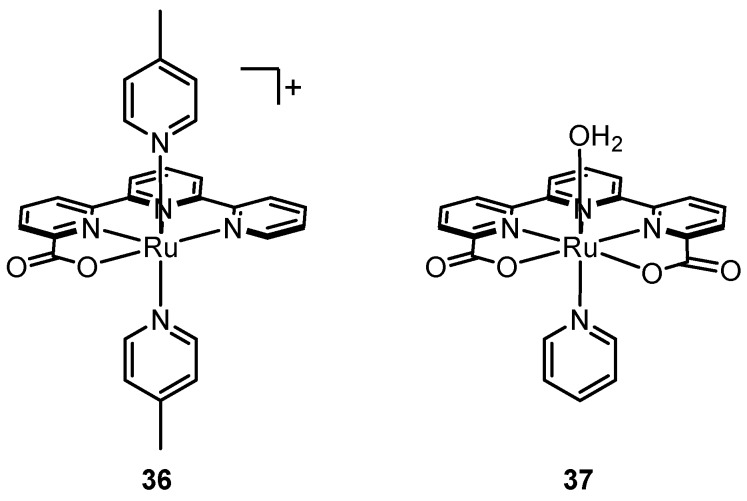
Variations of catalyst **35** that are significantly less catalytically active.

Llobet and coworkers reported [Ru^II^(tda-κ^4^-N_3_O)(py)] (**35**, tda = [2,2′:6′,2′′-terpyridine]-6,6′′-dicarboxylate), an astounding example demonstrating the power of internal bases (Figure 18) [102]. The ligand tda is reminiscent of bda but with an added pyridine to allow five potential binding sites. NMR spectroscopy of the Ru^II^ state of **35** shows symmetry in the peaks corresponding to tda, suggesting rapid fluxionality in metal-coordination of the two carboxylate groups on the NMR time scale. As shown by X-ray crystallography, upon oxidation to the Ru^IV^ state, the metal becomes electrophilic enough to form a [Ru^IV^(tda-κ^5^-N^3^O^2^)(py)_2_]^2+^ seven-coordinate species where both carboxylates are bound to Ru^IV^. Electrochemical data show that in neutral or basic aqueous solutions, a seven-coordinate [Ru^IV^(OH)(tda-κ^4^-N^3^O)(py)_2_]^+^ species is generated where de-coordination of a carboxylate group allows metal-coordination of a hydroxide. The [Ru^IV^(OH)(tda-κ^4^-N^3^O)(py)_2_]^+^ species undergoes PCET to form a Ru^V^=O species that is active for water oxidation via the WNA pathway. At pH 7, a large electro-catalytic wave is observed at an onset potential of ~1.2 V (vs. Ag/AgCl) for which the TOF_max_ was calculated to be ~8000 s^−1^, the highest reported thus far at neutral pH; for **27** the measured value was ca. 11 s^−1^ [103]. The free energy of activation (ΔG^ǂ^) calculated for O-O bond formation by WNA on the Ru^V^=O species concomitant with proton-transfer to the dangling carboxylate was significantly lower compared to other examples. Fan et al. reported a similar complex [Ru^II^(tpc)(pic)_2_]^+^ (**36**) (tpc = 2,2′:6′,2′′-terpyridine-6-carboxylate), which is almost structurally identical to **35** but does not have the additional dangling carboxylate (Figure 19). While TOF values were not calculated from electrochemical data of **36**, a qualitative comparison of cyclic voltammograms of **36** and **35** under similar conditions (pH ~10–11, phosphate buffer, 100 mV s^−1^ scan rate) clearly shows that the magnitude of the catalytic wave of **35** is substantially greater than that of **36**, thus giving empirical support to the ancillary role of the dangling carboxylate [102,104]. Further support is provided in a follow-up work by Matheu et al.; when one of the pyridine ligands of **35** is replaced by a water molecule to make the complex **37**, the TOF under the same conditions (pH 7) becomes nearly four orders of magnitude smaller [105]. Computations suggest that the active intermediate in the catalytic cycle of **37** is a seven-coordinate Ru^V^=O species where the oxo is axial and both carboxylates are bound. With the carboxylates occupied with metal-coordination and the axial oxo positioned further away, presumably intramolecular hydrogen bonding becomes inaccessible (or at least less accessible) during WNA and the free energy of activation (∆G^‡^) for O-O bond formation in **37** is raised by 5.2 kcal/mol compared to **35**. Intramolecular proton transfer has been invoked as a mechanism for catalysis for several other Ru complexes [106,107]. These results suggest that incorporation of Lewis bases and hydrogen bonding functionalities on the ligand near the active site is a highly promising strategy for increasing rates of water oxidation. 

### 2.12. Catalyst Modification Pathways

**Figure 20:**
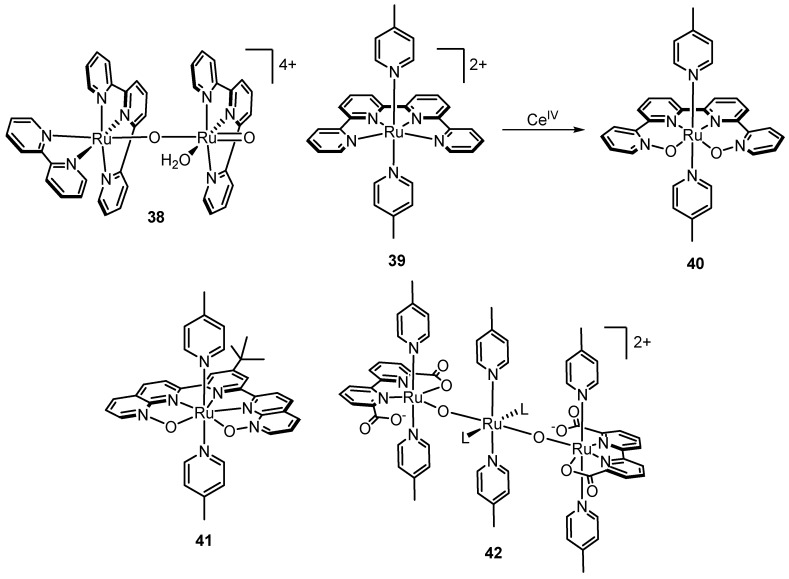
Various species derived from catalyst modification under oxidative conditions.

While Ru-polypyridyl complexes do generally tend to be quite robust, under the highly oxidative conditions required for water oxidation catalysis, they are not immune to modification pathways (Figure 20). Several research groups have reported on ligand *N*-oxidation in the context of water oxidation. In a thorough study of Ru(tpy)(bpy)-type complexes, workers in the Berlinguette group subjected [Ru(tpy)(bpy)](ClO_4_)_2_ to 1000 equiv of Ce^IV^ and after 24 h found NMR and ESI-MS evidence of 2,2′-bipyridine-N,N’-dioxide (in nearly ~50% yield) [70]. As a control, 2,2′-bipyridine alone stirred under the same conditions (1000 equiv of Ce^IV^, 1 M HClO_4_) did not lead to any N-oxide formation, suggesting involvement of the metal center in N-oxidation. Ahlquist computed a potential mechanism for *N*-oxidation of 2,2′-bipyrimidine in complex **23** which begins with substitution of the pyrimidine half *trans* to the oxo ligand with H_2_O (due to a strong *trans* effect) while the other half remains coordinated [108]. Then, multiple proton transfers between the coordinated water molecule and the oxo ligand lead to regeneration of an oxo ligand directly adjacent to the nitrogen of the unbound pyrimidine half of which is then primed for oxidation. *N*-oxidation could be invoked in a work by López et al., where it was found that exposure of [Ru(tpy)(bpy)(OH_2_)]^2+^ to oxidizing conditions (bulk electrolysis at 1.6 V vs. SSCE) led to loss of bpy to form a [Ru^VI^(tpy)(O)_2_(H_2_O)]^2+^ species, which then reacts with [Ru(tpy)(bpy)(OH_2_)]^2+^ to form µ-oxo dimer **38** [109]. Formation of **38** was evaluated by monitoring UV-visible and resonance Raman bands during bulk electrolysis and comparing with bands observed from synthetically prepared **38**.

Ligand loss does not necessarily lead to loss of catalysis; in fact, electrochemical data suggest that **38** is more robust and its catalytic ability is comparable to [Ru(tpy)(bpy)(OH_2_)]^2+^. Liu et al. monitored the reaction of [Ru(qtpy)(pic)_2_]^2+^ (**39**) with Ce^IV^ by ESI-MS and found that after addition of only 8 equiv of Ce^IV^, the parent peak for **39** at *m*/*z* = 299 completely disappeared and a peak at *m*/*z* = 315 corresponding to a [**39** + 2O]^2+^ species became predominant [110]. X-ray crystallography after precipitation by addition of NH_4_PF_6_ revealed complex **40** where two pyridines of qtpy are converted to *N*-oxides consistent with ESI-MS data. Interestingly, while no catalytic wave is seen in cyclic voltammetry of **39**, complex **40** shows onset of a catalytic wave at ~1.3 V (vs. SCE) suggesting that *N-*oxidation in fact activates catalysis, meaning that **39** is a pre-catalyst. Moonshiram et al. reported a similar phenomenon where the uncoordinated nitrogen atoms of the NPM ligand in Thummel’s complex **6b** rapidly undergo oxygen atom transfer to produce complex **41** bearing two *N-*oxide functionalities [111]. Despite the universal interest in polypyridyl ligands for WOC, it is clear from the above examples that one must be cautious, particularly in positioning pyridyl functionalities *cis* and hence closest to the active site. 

Ligand loss has also been observed in the context of the Ru(bda) systems. Nearly coincident to each other, Zhang et al. and Tsubonouchi et al. reported observation of **42** from oxidation of catalyst **27**. Zhang et al. found that **42** evolved after subjecting **27** to oxidizing equivalents of Ru(bpy)_3_Cl_2_/[Co(NH_3_)_5_Cl]Cl_2_ and NaIO_4_ in neutral media. Complex **42** was not a competent catalyst; however, the authors found that its formation from **27** could be reduced by lowering the pH. On the other hand, Tsubonouchi et al. found that trimer **42** could form simply from aerobic oxidation. Ligands bearing phenolate groups are prone to oxidative degradation and can convert completely to carboxylates, as has been shown by Kagalwala et al. and Liu et al. [112,113]. Clearly, further work is needed to identify more stable ligands to enable more stable catalysts. 

## 3. Conclusions

Molecular ruthenium catalysts have evolved significantly since the report of the “blue dimer” in 1982. Each iteration of ligand design brings new insight into structure-activity relationships. The early years (1980s–early 2000s) were dominated by the idea that at least two metal centers were required for catalysis; however, in 2004, Zong and Thummel’s report of Ce^IV^-induced water oxidation by complexes **6a**–**c** challenged this belief. In 2008, Meyer’s group conducted the first mechanistic investigation of mononuclear Ru catalysts (**23** and **24**) through E vs. pH studies and by monitoring changes in UV-visible spectroscopy upon addition of Ce^IV^. Debate about the mechanism of [Ru(tpy)(NN)(OH_2_)]^2+^ catalysts continues, and the role of the [Ru^V^=O] state is still not totally clear.

The benefits of tetradentate ligands were uncovered in Thummel’s systematic investigation of 19 complexes, which contained mixed combinations of pyridyl, bipyridyl, terpyridyl, and/or quaterpyridyl ligands. Turnover frequencies and numbers were the largest for **21** bearing the tetradentate dpp ligand, even though it is coordinatively saturated and contains no pre-coordinated water molecule. Thummel made the initial hypothesis that the wide *N*-Ru-*N* opening permitted coordination of a water molecule as a seventh-ligand to the metal center after oxidation to a coordinatively unsaturated 16 e^−^ Ru^IV^ species. In 2009, Sun’s group reported **27** containing 2,2′-bipyridine-6,6′-dicarboxylate, a tetradentate ligand with two anionic ligands. The influence of the anionic ligands marked as redox couples (Ru^II/III^, Ru^III/IV^) were observed at significantly more negative potentials compared to similar complex **21** with no anionic groups. Furthermore, addition of 2 equiv of Ce^IV^ to **27** afforded X-ray quality crystals of a pentagonal bipyramidal [Ru^IV^-OH] species lending support to Thummel’s hypothesis of coordination of water a seventh ligand. In 2010, Meyer demonstrated the influence of external bases on lowering energies of activation for O-O bond formation. 

In 2015, Llobet showed that a basic functionality built into the ligand architecture and positioned in the proximity of the catalytic active site could increase the efficiency of proton transfer and thus dramatically increase the rate of catalysis. Looking forward, such multi-functionality will likely be the key to efficient catalysis. In addition, ligands must be designed cautiously to avoid unwanted modification pathways. Thinking of catalysis as a race between the desired process (catalysis) and the undesired one (degradation), there are two ways to win the race: make a faster catalyst, or make a more stable one. No doubt, we will need both strategies to win the race.
